# Reduced Dietary Omega-6 to Omega-3 Fatty Acid Ratio and 12/15-Lipoxygenase Deficiency Are Protective against Chronic High Fat Diet-Induced Steatohepatitis

**DOI:** 10.1371/journal.pone.0107658

**Published:** 2014-09-24

**Authors:** Milos Lazic, Maria Eugenia Inzaugarat, Davide Povero, Iris C. Zhao, Mark Chen, Madlena Nalbandian, Yury I. Miller, Alejandra C. Cherñavsky, Ariel E. Feldstein, Dorothy D. Sears

**Affiliations:** 1 Department of Pediatrics, University of California San Diego, La Jolla, California, United States of America; 2 Institute of Immunology, Genetics and Metabolism, CONICET-UBA, Buenos Aires, Argentina; 3 Skaggs School of Pharmacy and Pharmaceutical Sciences, University of California San Diego, La Jolla, California, United States of America; 4 Department of Medicine, University of California San Diego, La Jolla, California, United States of America; State University of Rio de Janeiro, Biomedical Center, Institute of Biology, Brazil

## Abstract

Obesity is associated with metabolic perturbations including liver and adipose tissue inflammation, insulin resistance, and type 2 diabetes. Omega-6 fatty acids (ω6) promote and omega-3 fatty acids (ω3) reduce inflammation as they can be metabolized to pro- and anti-inflammatory eicosanoids, respectively. 12/15-lipoxygenase (12/15-LO) enzymatically produces some of these metabolites and is induced by high fat (HF) diet. We investigated the effects of altering dietary ω6/ω3 ratio and 12/15-LO deficiency on HF diet-induced tissue inflammation and insulin resistance. We examined how these conditions affect circulating concentrations of oxidized metabolites of ω6 arachidonic and linoleic acids and innate and adaptive immune system activity in the liver. For 15 weeks, wild-type (WT) mice were fed either a soybean oil-enriched HF diet with high dietary ω6/ω3 ratio (11∶1, HFH), similar to Western-style diet, or a fat Kcal-matched, fish oil-enriched HF diet with a low dietary ω6/ω3 ratio of 2.7∶1 (HFL). Importantly, the total saturated, monounsaturated and polyunsaturated fat content was matched in the two HF diets, which is unlike most published fish oil studies in mice. Despite modestly increased food intake, WT mice fed HFL were protected from HFH-diet induced steatohepatitis, evidenced by decreased hepatic mRNA expression of pro-inflammatory genes and genes involved in lymphocyte homing, and reduced deposition of hepatic triglyceride. Furthermore, oxidized metabolites of ω6 arachidonic acid were decreased in the plasma of WT HFL compared to WT HFH-fed mice. 12/15-LO knockout (KO) mice were also protected from HFH-induced fatty liver and elevated mRNA markers of inflammation and lymphocyte homing. 12/15-LOKO mice were protected from HFH-induced insulin resistance but reducing dietary ω6/ω3 ratio in WT mice did not ameliorate insulin resistance or adipose tissue inflammation. In conclusion, lowering dietary ω6/ω3 ratio in HF diet significantly reduces steatohepatitis.

## Introduction

Obesity has reached pandemic levels taking a significant toll on the health of adults and children. Obesity is associated with a range of metabolic maladies, including insulin resistance, dyslipidemia, nonalcoholic fatty liver disease (NAFLD) and hypertension, collectively grouped into the so-called metabolic syndrome [1], [2], [3]. Individuals with metabolic syndrome frequently exhibit pro-inflammatory metabolic profiles and have increased risk for developing type 2 diabetes and cardiovascular disease. The obesity-associated low-grade chronic inflammatory state in metabolic tissues, including adipose, liver, and muscle, contributes to insulin resistance and metabolic dysfunction that results from nutrient excess [4]. A variety of interventions to inhibit inflammatory pathways have been shown to have beneficial effects on metabolic function in rodent models of obesity and human obesity [4].

A new area of promising research in therapeutic approaches against obesity and related metabolic diseases is the use of anti-inflammatory nutrients provided through diet. The average Western-style diet is highly enriched in polyunsaturated omega-6 fatty acids (ω6) and relatively deficient in omega-3 fatty acids (ω3), reaching the estimated dietary ω6/ω3 ratio of 10–20∶1 [5]. Omega-6 and ω3 fatty acids are functionally distinct and their metabolites often have the opposing physiologic functions. While diets rich in ω6 are generally pro-inflammatory and promote insulin resistance, the converse is true for ω3-enriched diets [6]. Corresponding to Western diets, standard high fat (HF) diet chows in rodent diet-induced obesity models are typically enriched for saturated fatty acids and polyunsaturated ω6 fatty acids. HF chow consumption increases tissue percentage of saturated fatty acids and polyunsaturated ω6 fatty acids, decreases tissue percentage of ω3, and directly activates pro-inflammatory toll-like receptor 4 (TLR4) signaling [7], [8]. Thus, excessive amount of ω6 and a very high ω6/ω3 ratio in the diet are associated with and thought to be casual in the pathogenesis of many diseases, including cardiovascular disease, cancer and inflammatory and autoimmune diseases [5]. The reduction of ω6/ω3 ratio has also shown to be beneficial in the liver studies [9], [10]. Conversely, diets enriched with ω3 have been shown to reduce hypertriglyceridemia [11], fatty liver disease [12], [13], and improve renal function in type 2 diabetics [14].

Omega-6 and ω3 polyunsaturated fatty acids can be non-enzymatically oxidized and are also substrates for oxidation by lipoxygenases (LO), cyclooxygenases, and epoxygenases. In particular, 12/15-LO catalyzes the insertion of molecular oxygen into the ω6 arachidonic acid and linoleic acid; 12- and 15-hydroxy-eicosatetraenoic acids (HETE) are 12/15-LO products of arachidonic acid and 9- and 13- hydroxy-octadecadienoic acids (HODE) are 12/15-LO products of linoleic acid, all of which are pro-inflammatory. We have previously demonstrated that 12/15-LO knockout (KO) mice are protected from insulin resistance and adipose tissue inflammation induced by a short-term feeding with ω6-enriched HF diet [15].

In this study we aimed at investigating the long-term effects of altering dietary ω6/ω3 ratio in HF diet induced obesity in wild-type (WT) mice with focus on the liver and adipose tissue inflammation. We gave careful consideration on the design of the diets so that the amount of total saturated, monounsaturated and polyunsaturated fatty acids was the same in each HF diet. Moreover, the total amount of dietary fat, the ratios of ω6 to ω3, and the amount of total eicosapentaenoic acid (EPA) and docosahexaenoic acid (DHA) in our low ω6/ω3 ratio diet are more relevant to the ratios and doses used in human studies which are significantly lower in comparison to the majority of published rodent studies investigating the anti-inflammatory effects of ω3-supplementation [16], [17], [18], [19], [20]. We assessed the circulating concentration of oxidized metabolites of pro-inflammatory ω6 arachidonic and linoleic fatty acids and examined markers of both innate and adaptive immune systems in liver tissue. Lastly, we sought to delineate the effects of 12/15-LO deficiency on hepatic steatosis in the chronic ω6-enriched diet obese rodent model.

## Experimental Procedures

### Animal studies

**Figure 1:**
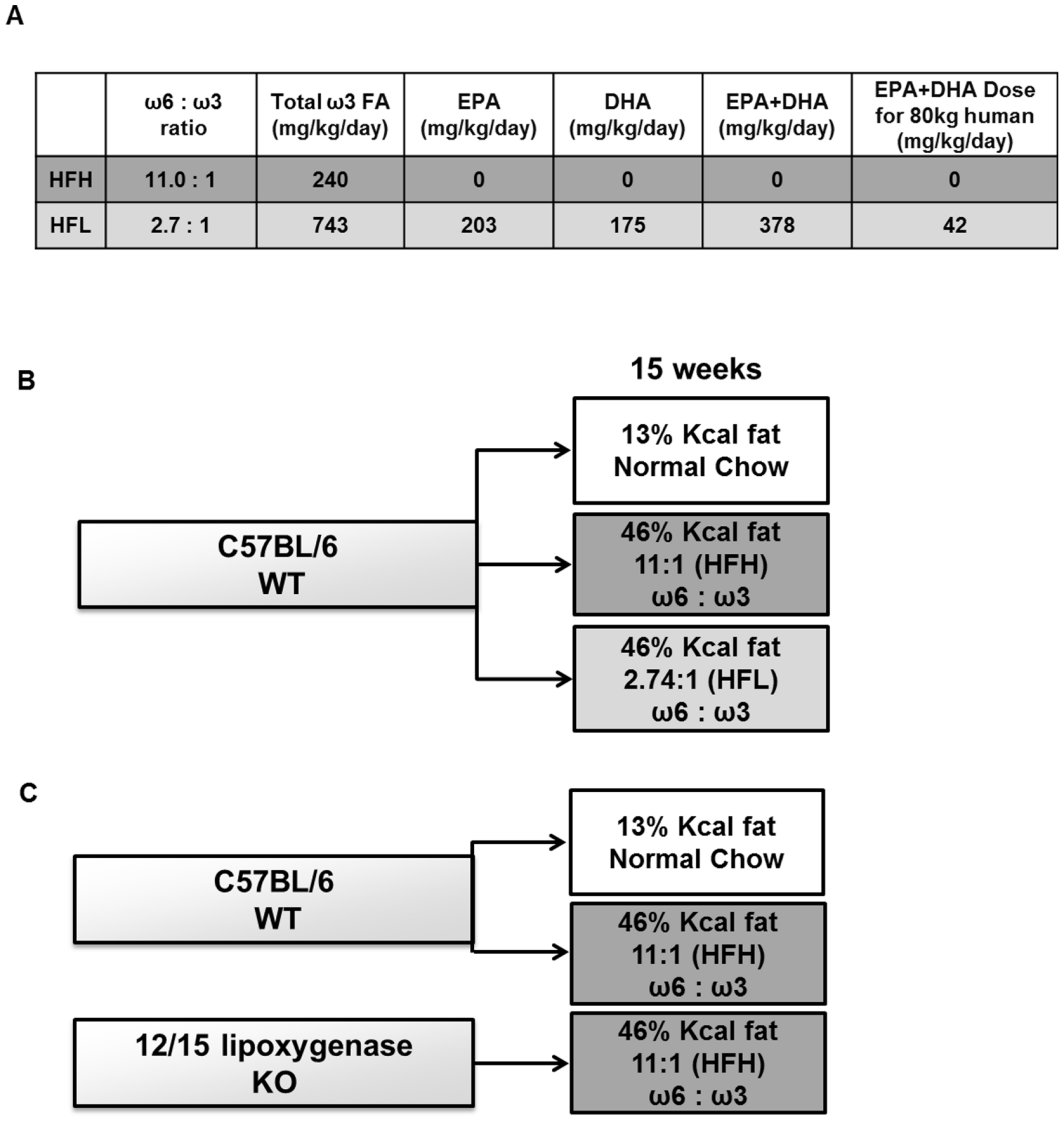
Schematic of diets and mouse groups. (A) Schematic illustrates the dietary ω6/ω3 fatty acid ratio, and the mg/kg/day intake of total ω3 fatty acid, EPA, DHA and EPA+DHA in the HFH and HFL diets consumed by the mice. For comparison, the approximate mg/kg/day dose of EPA+DHA for an 80 kg human taking a standard prescription of Lovaza fish oil (four 1 g capsules, containing 840 mg EPA+DHA each, per day) is shown. (B) 15-week diet regimens for WT mice: normal chow diet (13% Kcal from fat), high fat diet (46% kcal from fat) with high dietary ω6/ω3 ratio (HFH), or high fat diet (46% kcal from fat) with low dietary ω6/ω3 (HFL). (C) 15-week diet regimens for WT and 12/15-LOKO mice: high fat diet (46% kcal from fat) with high dietary ω6/ω3 ratio (HFH). FA, fatty acids; EPA, eicosapentaenoic acid; DHA, docosahexaenoic acid.

All mouse procedures were performed in accordance with the *Guide for Care and Use of Laboratory Animals* of the National Institutes of Health and were approved by the University of California, San Diego, Animal Subjects Committee (protocol #S99173). All efforts were made to minimize pain and distress during animal husbandry and experimental assessments. Fourteen- to 15-week old male C57BL/6J WT mice (Jackson Laboratory, Bar Harbor, ME) were fed with normal control (NC, n = 7) rodent diet Purina 5001 (13% calories from fat; LabDiet, St. Louis, MO), high fat diet with high dietary ω6/ω3 ratio of 11∶1 (HFH, n = 7; 46% calories from fat), representing typical Western diet, or high fat diet with low dietary ω6/ω3 ratio of 2.7∶1 (HFL, n = 8; 46% calories from fat), Table 1. Both HF diets were custom designed and manufactured by TestDiet (PMI Nutrition International, St. Louis, MO) using their basic van Heek standard obesogenic diet 58V8. The HFH fat components were lard and soybean oil, the HFL fat components were lard, soybean oil, and Menhaden fish oil. Care was taken to ensure palatability and prevent reduced calorie intake in the HFL-fed mouse group, challenges that have been previously reported in other fish oil/ω3-enriched diet studies [19], [20]. Fish oil fragrance can lead to reduced food intake, so both high fat diets were prepared with bacon flavoring and anti-oxidant t-butylhydroquinone (0.002% wt/wt) in order minimize potential odor-effects on food intake with the HFL diet. Both HFL and HFH diets were developed in pelleted form. Fifteen-week old male 12/15-LOKO mice (from our in-house colony [15] were fed with HFH diet (n = 4). Mice were housed under controlled light (12∶12 light: dark) and climate conditions with unlimited access to food and water. Mouse weights and food intake were monitored daily for one week and then twice a week for the next 9 weeks on respective diets. All mice were sacrificed after 15–17 weeks into the respective diets to assess liver pathophysiology and inflammatory gene expression, plasma components, gonadal white adipose tissue (gWAT) weight and macrophage infiltration. Mouse sacrifice was performed in a matched way so that the average length of the feeding period was the same in each group. Figure 1 shows the daily intake of total ω3 FA and EPA+DHA for the mice in our study and a schematic of the study groups. For comparison with the mouse daily dose in our study, Figure 1 also shows an approximate EPA+DHA daily dose commonly prescribed to an 80 kg human.

**Table 1:** Composition of diets.

	HFH	HFL
**Omega-6**	15.9%	12.5%
LA	15.7%	12.2%
AA	0.2%	0.3%
**Omega-3**	1.4%	4.6%
ALA	1.4%	1.1%
EPA+DHA	0.0%	3.5%
**ω6/ω3 ratio**	11	2.7
**SFA**	40.8%	41.5%
**MUFA**	42.0%	41.4%
**PUFA**	17.3%	17.1%
**Total fat**	46.3%	46.3%
**Protein**	18.2%	18.2%
**Carbohydrate**	35.5%	35.5%

Fatty acid subgroup values are represented as a percent of total fat. Total fat, protein, carbohydrate values are represented as a percent of total kcal of diets. HFH, high dietary ω6/ω3 ratio; HFL, low dietary ω6/ω3 ratio; LA, linoleic acid; AA, arachidonic acid; ALA, alpha-linoleic acid; EPA, eicosapentaenoic acid; DHA, docosahexaenoic acid; SFA, saturated fatty acids; MUFA, monounsaturated fatty acids; PUFA, polyunsaturated fatty acids.

### Assessment of Insulin Resistance by HOMA-IR

Homeostatic model assessment of insulin resistance (HOMA-IR) was calculated as fasting plasma insulin (µU/mL) × fasting blood glucose (mg/dL) ÷405] [21]. Plasma insulin levels were measured using the Insulin Ultrasensitive (Mouse) EIA method (Alpco, Salem, NH). A single plasma sample with insulin level outside of the linear range of the assay was been excluded from analysis. Fasting whole blood glucose was measured using a hand-held glucometer (OneTouch Ultra2, LifeScan, Inc. Milpitas, CA).

### Histology and the Assessment of Hepatic Steatosis in Mouse Livers

Livers were weighed, cut into small pieces and fixed in 10% formalin for 24 h or freshly frozen in Optimal Cutting Temperature compound (OCT). The formalin fixed tissues were embedded in paraffin and sections of 4 µm thickness were prepared. Haematoxylin and eosin stained sections were used for histopathological evaluation of hepatic injury and Oil Red O-stained frozen liver specimens was performed by the UCSD Histology Core. Samples were analyzed by light microscopy and the level of triglycerides assessed by ImageJ software (National Institutes of Health, Bethesda, MD). All photos were taken by NanoZoomer 2.0HT Slide Scanning System (Hamamatsu, Japan).

### Flow cytometry

Adipose tissue stromal vascular cells (SVCs) were isolated and analyzed by FACS as previously reported [22] with minor modifications. Briefly, freshly harvested epididymal fat pads were separately rinsed and minced in DPBS +1% BSA then treated with 1 mg/mL type II collagenase (Sigma) for 25 min in a 37°C shaking water bath. Adipose tissue cell suspensions were filtered through 100 µm mesh. SVCs were separated from floating adipocytes by centrifugation, incubated in RBC lysis buffer (eBioscience) for 5 min, then re-suspended in fresh DPBS +1% BSA. SVCs were incubated with Fc Block (BD Biosciences) for 15 min and then stained for 30 min with fluorescent-conjugated antibodies against F4/80 (Ab Serotec), CD11b (BD Biosciences), and CD11c (BD Biosciences). Cells were washed two times and re-suspended in DPBS +1% BSA and propidium iodide (Sigma). Presence of the fluorescent stains in the SVCs was analyzed using a FACS Calibur flow cytometer (BD Biosciences). Control SVCs preparations, including unstained cells, PI-only stained cells, and fluorescence-minus-one (FMO) stained cells, were used to set gatings and compensation. Total macrophages were defined as F4/80^+^CD11b^+^ cells, while M1-type macrophages were defined as F4/80^+^CD11b^+^CD11c^+^ cells.

### Detection and quantification of oxidized fatty acid profile

All plasma samples for analyses contained anti-oxidant cocktail [DTPA (2 mM final) and butylated hydroxytoluene (500 µM final)]. Levels of multiple fatty acid oxidation products (free plus esterified) in plasma were quantified using liquid chromatography online electrospray ionization tandem mass spectroscopy. Preparation and analysis of plasma samples was performed as previously reported [23]. The lipid peroxidation products analyzed included structurally specific species of HETE (5-, 8-, 9-, 11-, 12-, and 15-HETE), HODE (9- and 13-HODE), oxo-octadecadienoic acids (9- and 13-oxoODE) and their precursors arachidonic acid and linoleic acid.

### Gene expression

Liver tissue was preserved in RNA*later* (Invitrogen, Carlsbad, CA) and homogenized using the FastPrep 24 bead homogenization system (MP Biomedicals, Santa Ana, CA). Total RNA was isolated using RNeasy kit (Qiagen, Valencia, CA) and reverse transcribed by SuperScript III First-Strand Synthesis System RT-PCR Kit (Invitrogen) using oligodT primers according to the manufacturer instructions. The concentration and purity of RNA was assessed by NanoDrop (Thermo Scientific, Waltham, MA). The 260/280 ratio of each sample was at or slightly above 2. Additional on column DNAse digestion step was performed, and ‘no template’ and ‘no reverse transcriptase’ controls were run to ascertain purity of reagents and the lack of genomic DNA contamination, respectively. Individual samples were measured in triplicate. Data were normalized against two reference housekeeping genes: beta-2 microglobulin (B2m) and hydroxymethylbilane synthase (Hmbs) which were chosen based on stable gene expression levels, based on our pilot study. Others have also found a stable invariant expression of these genes in the livers of other high fat diet-induced obesity mouse models and hepatic cells [24], [25], [26]. The PCR primers used to amplify each gene are: B2m F-5′-CCCCACTGAGACTGATACATACG-3′ R-5′-CGATCCCAGTAGACGGTCTTG-3′, Ccr7 F- 5′-GTACGAGTCGGTGTGCTTCAA-3′ R-5′-GGTAGGTATCCGTCATGGTCT-3′, Ccl19 F- 5′-GTGATGGAGGGGTCAGGA-3′ R-5′-GGGATGGGACAGCCTAAACT-5′, Ccl21 F-5′-GTGATGGAGGGGGTCAGGA-3′ R-5′-GGGATGGGACAGCCTAAACT-3′, Cxcl1 (KC) F- 5′-CTGGGATTCACCTCAAGAACATC-3′ R-5′-CAGGGTCAAGGCAAGCCTC-3′, Cxcl2/3 (Mip2) F-5′-CCACCAACCACCAGGCTAC-3′ R-5′-GCTTCAGGGTCAAGGGCAAA-3′, Hmbs F-5′-TGTGGTGGCGATGCTGAAA-3′ R-5′-TTGTCTCCCGTGGACATA-3′, Ifng F-5′-ATGAACGCTACACACTGCATC-3′ R-5′-CCATCCTTTTGCCAGTTCCTC-3′, Il10 F-5′-GCTCTTACTGACTGGCATGAG-3′ R-5′-CGCAGCTCTAGGAGCATGTG-3′, Il12a F-5′-CTGTGCCTTGGTAGCATCTATG-3′ R-5′-CGCAGAGTCTCGCCATTATGAT-3′, Il12b F-5′-CTCAGAAGCTAACCATCTCCTGG-3′ R-5′-CACAGGTGAGGTTCACTGTTTC-3′, Il18 F-5′-GACTCTTGCGTCAACTTCAAGG-3′ R-5′-CAGGCTGTCTTTTGTCAACGA-3′, Srebp1 F-5′-GCAGCCACCATCTAGCCTG-3′ R-5′-CAGCAGTGAGTCTGCCTTGAT-3′, Tnfa F-5′-CCCTCACACTCAGATCATCTTCT-3′ R-5′-GCTACGACGTGGGCTACAG-3′. Quantitative Real-time PCR was performed on a CFX96 RT-PCR Detection System (BioRad, Hercules, CA, USA) by using Brilliant II SYBR Green QPCRMaster Mix (Agilent Technologies, La Jolla, CA, USA). Fold-change values were calculated using the 2^−ΔΔCt^ method.

### Data analysis

Repeated-measures one-way ANOVA was applied to body weight with a Bonferroni post hoc test to determine significant group differences over time. Two-tailed Student's t-test was used to analyze statistical significance between groups, as indicated in the figure legends. Data are reported as mean ± standard error of the mean (SE), with significance at *P*<0.05, unless otherwise noted. GraphPad software (La Jolla, CA) was used to perform analyses and to construct graphs.

## Results

### Lowering dietary ω6/ω3 increases food intake, but does not significantly alter total body, liver or adipose tissue weights in high fat diet induced obesity

**Figure 2:**
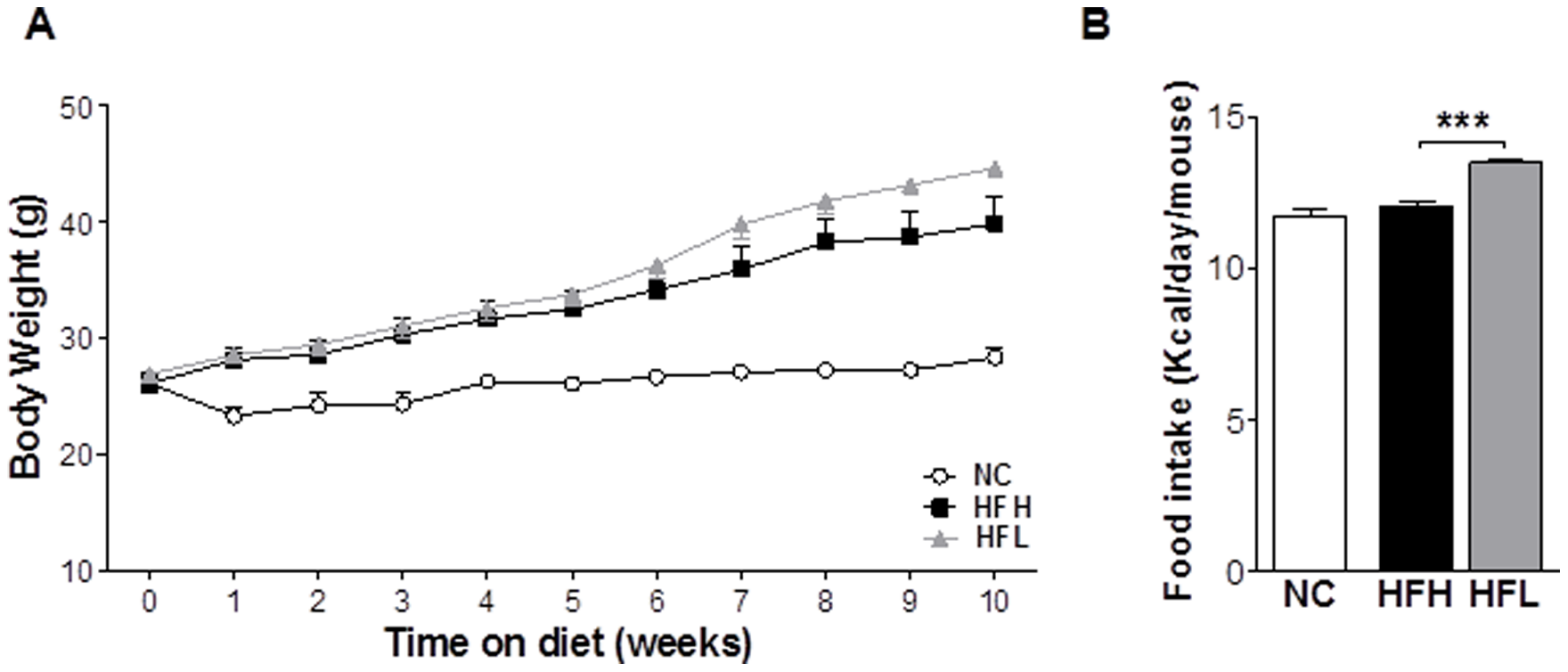
Body weight and food intake. The effect of varying dietary ω6/ω3 ratio in WT (NC n = 4), open circles; HFH n = 7, black squares; and HFL n = 8, gray triangles) mice on (A) body weight over fist 10 weeks of diet regimens and (B) food intake averaged over weeks 5 to 10 on the respective diets. Values are mean ±SE. ***P<0.001 HFH vs. HFL. NC, normal control (chow); HFH, high dietary ω6/ω3 ratio; HFL, low dietary ω6/ω3 ratio.

To assess the metabolic effects of altering dietary ω6/ω3 ratio in the context of a high fat diet, we fed WT mice isocaloric diets (46% kcal from fat) containing polyunsaturated fat contributed primarily from soybean oil (high ω6/ω3 ratio, HFH) or fish oil (low ω6/ω3 ratio, HFL), or low fat, normal chow (NC; 13% kcal from fat) (Fig. 1A and B). The high fat diets (HFH and HFL) were carefully designed to contain equivalent fat, protein and carbohydrate content as well as equivalent total saturated, monounsaturated, and polyunsaturated fatty acid content (Table 1). Both high fat diets induced significant body weight gain past week 5 as compared to NC (Fig. 2A). There was no significant difference in weight gain over the period of the first 10 weeks or in the final body weights (week 15) between WT mice fed HFH and HFL diets (Fig. 2A and Table 2). Although WT mice on HFL diet consumed significantly more calories daily, averaging over the period of the week 5 to 10 of the study (P<0.001) (Fig. 2B), increased food intake in the HFL group did not differentially affect liver or gonadal white adipose tissue (gWAT) weight as a percent of total body weight (Table 2). Finally, there were no differences in fasting plasma insulin and glucose levels, HOMA-IR, or proinflammatory M1-type macrophage content in gWAT between WT mice fed HFH and HFL diets (Table 2).

**Table 2:** Metabolic parameters in WT mice fed NC, HFH, and HFL diets.

	NC	HFH	HFL	
	mean±SE	mean±SE	mean±SE	t-test^b^
Parameter [unit]	(n = 3–7)^a^	(n = 6–7)	(n = 7–8)	p-value
Final body weight (BW) [g]	29.1±0.8	41.0±2.6	45.3±0.8	0.12
Liver weight [% BW]	5.7±0.3	5.0±0.3	5.2±0.4	0.67
gWAT weight [% BW]	2.3±0.3	4.4±0.3	4.1±0.1	0.50
Fasting plasma insulin [ng/mL]	0.30±0.11	1.82±0.56	2.62±0.56	0.34
Fasting blood glucose [mg/dL]	111±8	128±20	158±12	0.21
HOMA-IR	2.0±0.8	16.0±7.4	24.0±4.1	0.33
gWAT M1-type macrophages [% total macrophages]	18.9±1.7	80.6±7.0	89.3±1.0	0.21

NC, normal chow; HFH, high dietary ω6/ω3 ratio; HFL, low dietary ω6/ω3 ratio; HOMA-IR, homeostatic model assessment of insulin resistance; gWAT, gonadal white adipose tissue.

a Data were not available for liver weight, fasting insulin and glucose for 4 NC mice.

b Student's t-test was restricted to high fat diet groups (HFH vs. HFL). NC data are shown for comparison.

### Lowering dietary ω6/ω3 reduces lymphocyte homing response in the liver and decreases hepatic inflammation and steatosis

**Figure 3:**
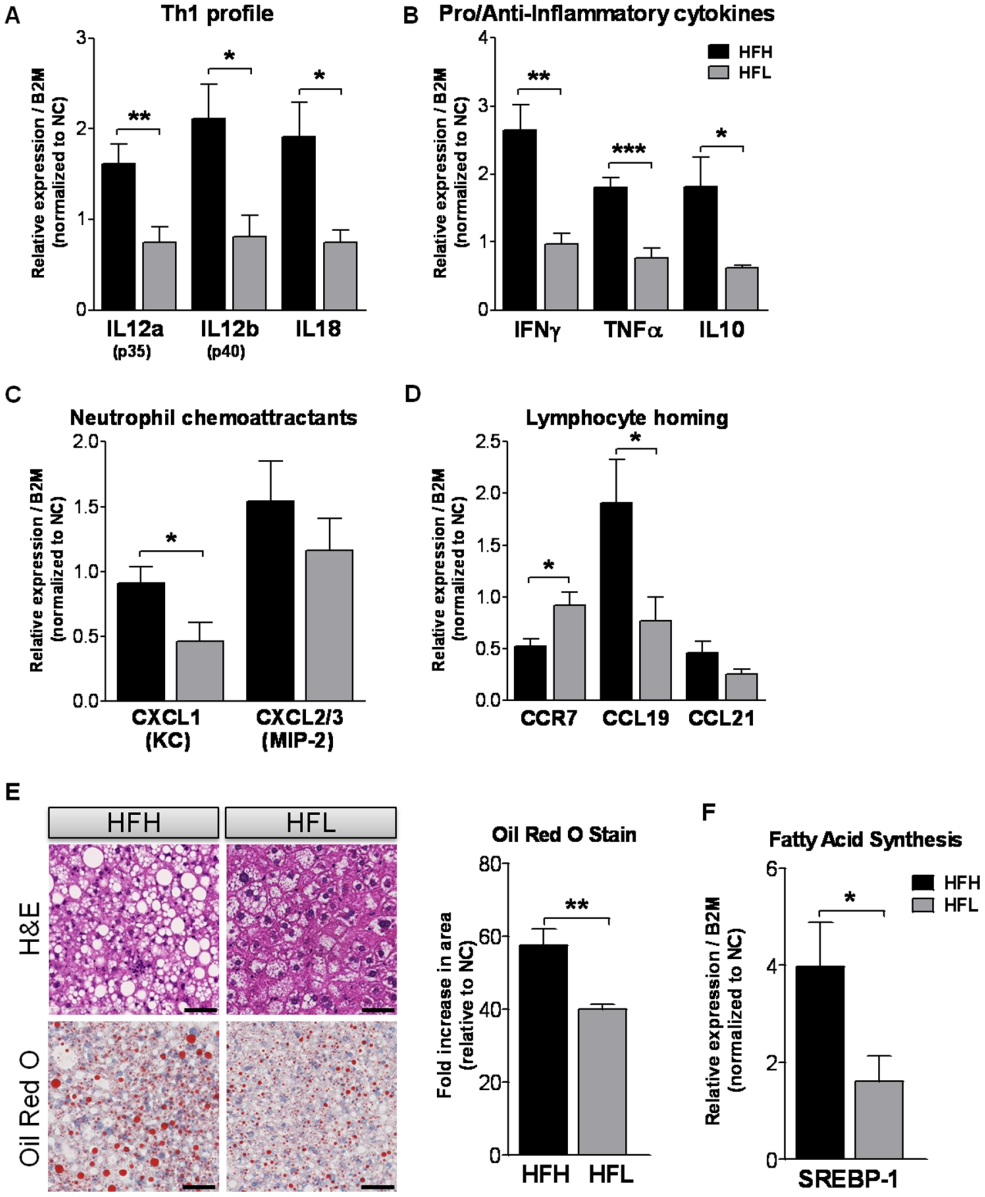
Assessment of hepatic inflammation and steatosis. The effect of varying dietary ω6/ω3 ratio in high fat diet-fed WT mice on the hepatic mRNA expression of (A) cytokines driving a Th1 profile, (B) pro/anti-inflammatory cytokines, (C) neutrophil chemoattractants, and (D) regulators of lymphocyte homing response, all normalized to signal in WT NC mice (n = 7). (E) Haematoxylin-eosin and Oil red O staining of paraffin-embedded liver sections. Quantitative grading scale assessing the quantity of hepatic triglycerides, normalized to signal in WT NC mice (expressed as a fold increase in stained area relative to WT NC mice). (F) The effect of varying dietary ω6/ω3 ratio in high fat diet-fed WT mice on the hepatic mRNA expression of sterol regulatory element-binding protein 1 (Srebp1) normalized to signal in WT NC mice. WT HFH: black bars; WT HFL: gray bars. Values are mean ±SE. 5-7 animals per group. *P<0.05, **P<0.01, ***P<0.001 HFH vs. HFL. Scale bar, 50 µm. WT NC SEs for genes Il12a (p35), Il12b (p40), Il18, Ifng, Tnfa, Il10, Cxcl1 (KC), Cxcl2/3 (Mip2), Ccr7, Ccl19, Ccl21 and Srebp1 were 0.14, 0.09, 0.14, 0.08, 0.12, 0.17, 0.14, 0.13, 0.20, 0.21, 0.15, and 0.10, respectively. WT NC SE value for oil red O stain was 0.15. NC, normal control (chow); HFH, high dietary ω6/ω3 ratio; HFL, low dietary ω6/ω3 ratio.

As it was previously reported that ω3 supplemented diet reduces hepatic steatosis and inflammation [18], [19], we next aimed to characterize these parameters in our animal models by measuring gene expression in liver tissue from the mouse groups. First we assessed the stimulation of Th1-mediated immune responses which generally lead to a pro-inflammatory phenotype and increased abundance of effectors IFN-γ and TNF-α. We found that HFL diet significantly reduced the mRNA levels of Il12a and b (p35 and p40 subunits) (P<0.05) and Il18 (P<0.05) as compared to HFH, thus marking attenuation of the inflammatory Th1 phenotype (Fig. 3A). Next, we assessed hepatic mRNA expression of representative pro-inflammatory (Infg and Tnfa) and anti-inflammatory pathway (Il10) genes and neutrophil chemoattractant genes, C-X-C motif ligand 1 (Cxcl1, KC) and microphage inflammatory protein 2 (Mip2; Cxcl2/3). HFL diet had a protective, anti-inflammatory effect where levels of Ifng (P<0.01) and Tnfa (P<0.001) mRNA were near to normal chow (NC) control levels, as compared to the effect of HFH diet (Fig. 3B). Levels of Il10 and Cxcl1 mRNA were both significantly lower in HFL vs. HFH diet-fed mice (P<0.05) (Fig. 3C). Next, the lymphocyte homing response was examined. When chemokine C-C motif ligands (CCL) 19 or 21 bind to lymphocyte-expressed chemokine receptor (CCR) 7, this receptor is internalized and lymphocytes stay trapped in an organ. Indeed, it has been shown that the suppression of cytokine signaling protein-1 regulates migration of CD4^+^ T cells into peripheral tissues via changes in Ccr7 expression levels [27]. Accordingly, we observed a decrease in the expression of Ccr7 (P<0.05) and increase in the expression of Ccl19 (P<0.05) in HFH-fed animals, a phenotype that did not exist in HFL diet-fed mice, suggesting that lower dietary ω6/ω3 ratio in the context of a high fat diet protects from lymphocyte homing in the liver (Fig. 3D). Interestingly, HFL diet did not alter high fat diet-induced infiltration of M1-type inflammatory macrophages into gWAT, as determined by flow cytometry analysis (Table 2). Histological analysis of H&E and Oil Red O stained liver specimens demonstrated significantly less hepatic steatosis in HFL as compared with HFH diet-fed mice (Fig. 3E). Correspondingly, the expression level of the sterol regulatory element-binding protein 1 gene (Srebp1) was significantly lower in HFL vs. HFH diet fed mice (P<0.05) (Fig. 3F).

### Oxidized metabolites of arachidonic acid are significantly decreased in the plasma of HFL diet-fed mice

**Figure 4:**
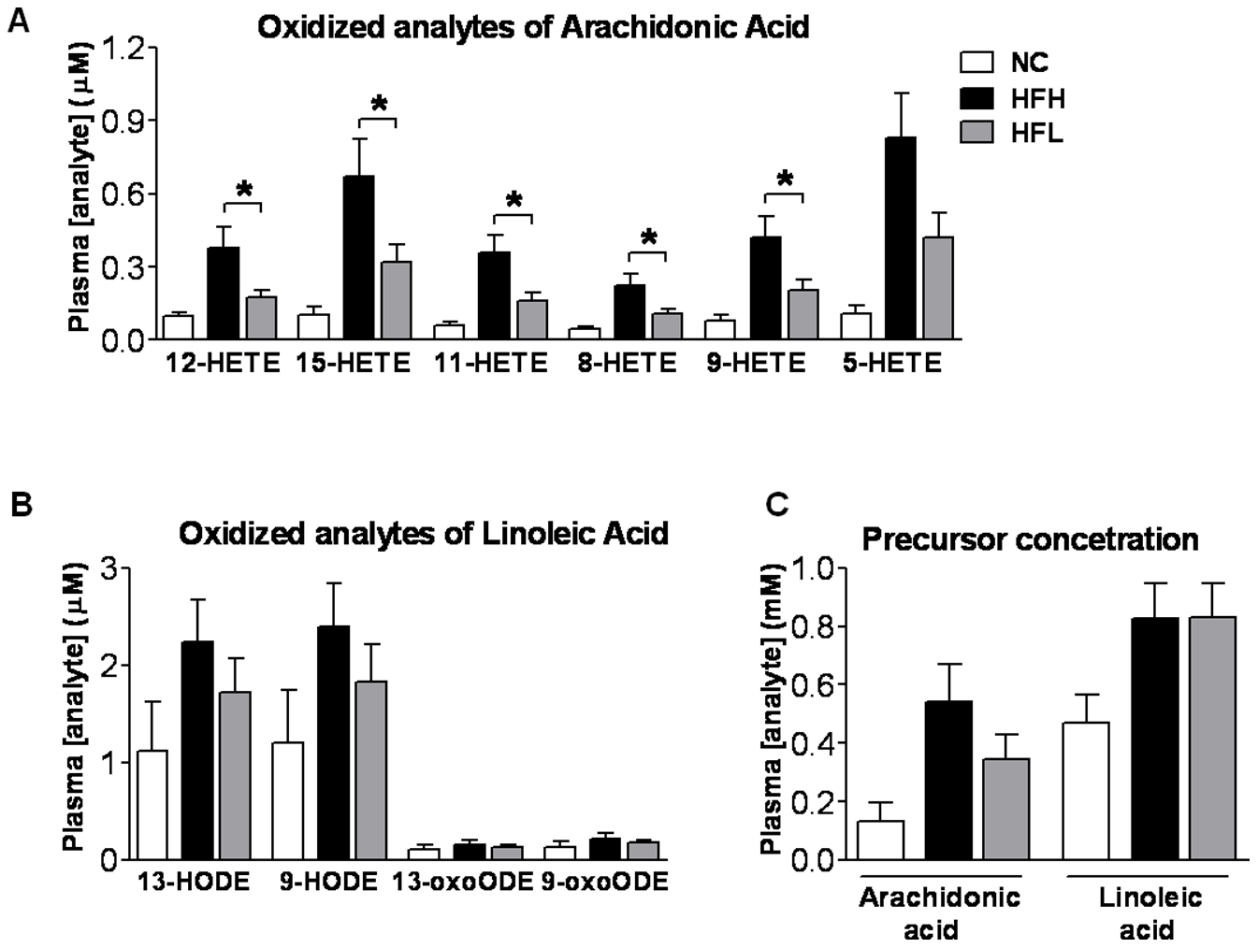
Plasma concentration of the major ω6 fatty acids and their corresponding pro-inflammatory metabolites. The effect of varying dietary ω6/ω3 ratio in high fat diet-fed WT mice on plasma concentrations of oxidized metabolites of (A) arachidonic acid and (B) linoleic acid. (C) Plasma concentrations of arachidonic and linoleic acids. NC n = 3 (open bars), HFH n = 7 (black bars), HFL n = 8 (gray bars). T-test analysis was restricted to HF diet animals only. Values are mean ±SE. *P<0.05 HFH vs. HFL. NC, normal control (chow); HFH, high dietary ω6/ω3 ratio; HFL, low dietary ω6/ω3 ratio; HETE, hydroxyeicosatetraenoic acid; HODE, hydroxyoctadecadienoic acid; oxoODE, oxooctadecadienoic acid.

We have shown previously that 9- and 13-HODEs and 9- and 13-oxoODEs, products of free radical and enzymatic lipoxygenase-mediated oxidation of the ω6 linoleic acid were significantly elevated in patients with non-alcoholic steatohepatitis (NASH) [14]. Plasma concentrations of 12-, 15-, 11-, 8- and 9-HETE oxidized metabolites of the ω6 arachidonic acid were significantly lowered in HFL vs. HFH diet fed mice (all P<0.05) (Fig. 4A). There were no significant changes in the plasma concentrations of oxidized metabolites of linoleic acid (HODEs and oxo-ODEs) or ω6 precursor concentrations of plasma arachidonic acid or linoleic acid (Fig. 4B and C).

### 12/15-lipoxygenase deficiency prevents metabolic defects and liver abnormalities associated with high fat, high ω6/ω3 ratio diet

**Figure 5:**
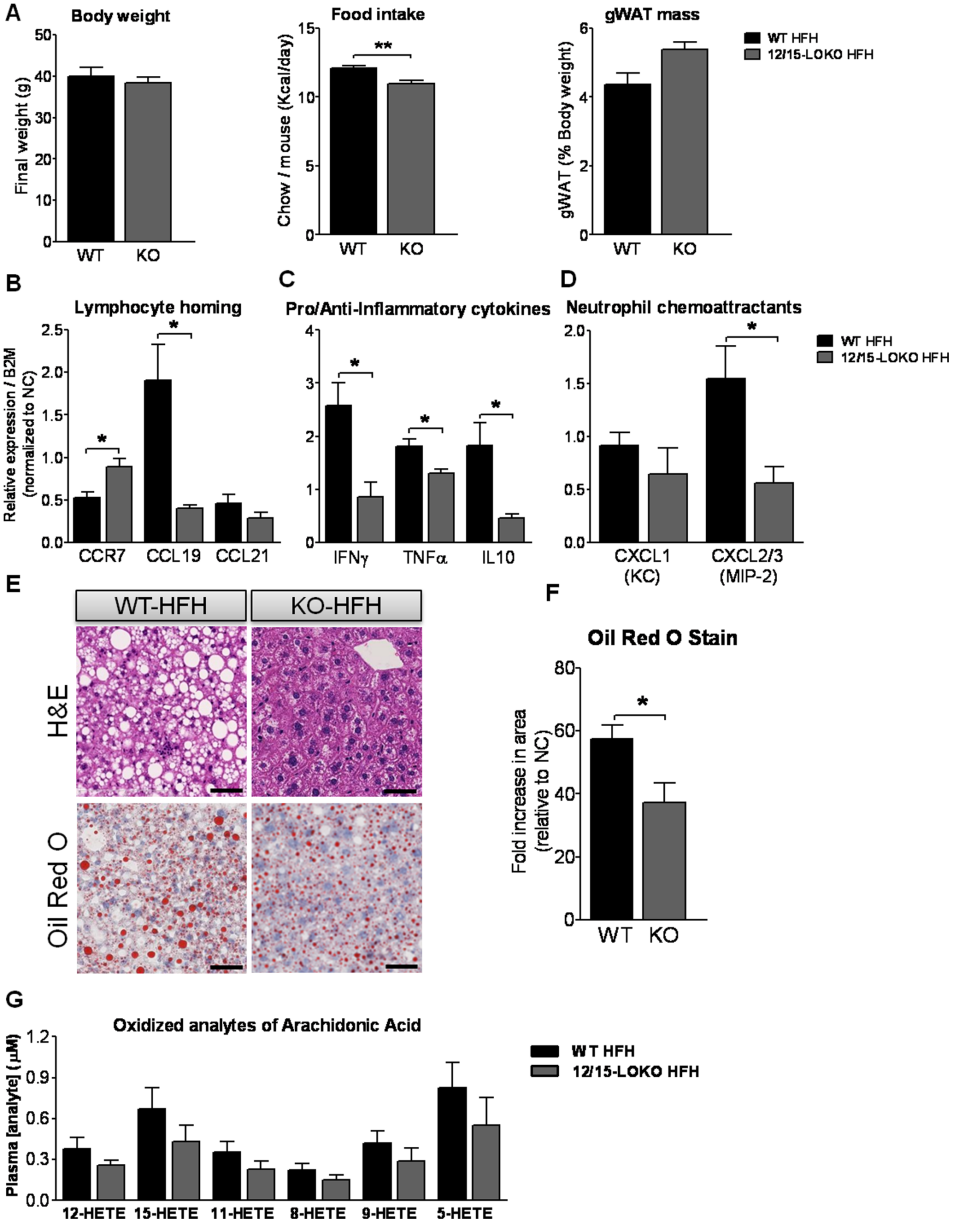
Assessment of hepatic inflammation and steatosis in 12/15-LOKO mice on HFH diet. The effect of 12/15-lipoxygenase deficiency on (A) terminal body weight, food intake and gonadal white adipose tissue (gWAT) weight expressed as percent total body weight, and hepatic expression of (B) regulators of lymphocyte homing response, (C) pro/anti-inflammatory cytokines and (D) neutrophil chemoattractants. (E) Haematoxylin-eosin and Oil red O staining of paraffin-embedded liver sections to show hepatic steatosis. (F) Quantitative grading scale assessing the quantity of hepatic triglycerides, normalized to signal in WT NC (n = 7) mice (expressed as a fold increase in stained area relative to WT NC mice). (G) Plasma concentrations of oxidized metabolites of arachidonic acid in KO HFH n = 4 (dark gray bars) compared to WT HFH n = 7 (black bars) mice. Values are mean ±SE. *P<0.05, **P<0.01 WT HFH vs. 12/15-LOKO HFH. Scale bar, 50 µm. WT NC SEs for genes Ccr7, Ccl19, Ccl21, Ifng, Tnfa, Il10, Cxcl1 (KC), Cxcl2/3 (MIP-2) were 0.20, 0.21, 0.15, 0.08, 0.12, 0.17, 0.14, 0.13, respectively. WT NC SE value for oil red O stain was 0.15. KO, 12/15 lipoxygenase knockout; HFH, high dietary ω6/ω3 ratio; gWAT, gonadal white adipose tissue; HETE, hydroxyeicosatetraenoic acid.

We and others have previously shown that 12/15-LOKO mice are protected from HF diet-induced insulin resistance and adipose tissue inflammation [15], [28]. In this study, we were interested in additionally studying the liver phenotypes of 12/15-LOKO mice after HF feeding. 12/15-LOKO mice were subjected to HFH diet for the same duration as WT mice and compared to WT HFH mice to assess whether 12/15-lipoxygenase deficiency prevents metabolic defects associated with high fat, high ω6/ω3 ratio diet (Fig. 1C). There were no significant differences in the final total body weights or gWAT mass expressed as a percentage of total body weight among any of the groups (Fig. 5A) even though the KO mice consumed significantly less calories daily, averaged over weeks 5 to 10 of the study (P<0.01) (Fig. 5A). Gene expression markers of lymphocyte homing response in liver were reduced (Fig. 5B), as was expression of hepatic cytokine and chemoattractant genes (Ifng, Tnfa, Il10 and Cxcl2/3) in KO HFH as compared to WT HFH (all P<0.05) (Fig. 5C and D) similar to that observed in the WT HFL group (Fig. 3B and C). In addition, hepatic steatosis was also significantly less in the KO HFH as compared to WT HFH mice (Fig. 5E and F). Although plasma levels of oxidized metabolites of arachidonic acid were not individually significantly reduced in the KO mice (Fig. 5G), they were all trending lower as expected given that 12/15-lipoxygenase is an enzymatic source of 12- and 15-HETE. Levels of oxidized arachidonic acid metabolites of in the KO mice approximate those observed in the HFL-fed WT mice (Fig. 4A).

## Discussion

Nonalcoholic fatty liver disease has become the most common liver disease in the United States and other developed countries. It is commonly associated with the metabolic syndrome, obesity, type 2 diabetes and dyslipidemia. With the addition of inflammation, NAFLD can progress to a more serious condition termed NASH which then can progress to cirrhosis and hepatocellular carcinoma [29]. Common causes of NAFLD, such as Western diet-induced obesity and insulin resistance, are associated with macrophage infiltration and inflammation in adipose, liver and muscle tissues of humans and rodent models where a feed-forward cycle of reciprocal adipocyte, hepatocyte, myocyte and macrophage cross-talk results in the secretion of inflammatory factors and further macrophage recruitment [4], [30], [31], [32]. Thus, reducing this chronic inflammation may be the key to combating obesity-induced morbidities, insulin resistance and NAFLD. We aimed to identify new strategic dietary approaches for mitigating HF diet-induced inflammation and expand our knowledge of 12/15-LO activity. This study is the first to show that reducing dietary ω6/ω3 fatty acid ratio, without any other diet composition changes, is sufficient to protect WT mice from long-term HF diet-induced steatohepatitis and to reduce plasma concentration of oxidized metabolites of arachidonic acid. This reduction in dietary ω6/ω3 ratio did not protect the WT mice from HF diet-induced insulin resistance or adipose tissue inflammation. In addition, we demonstrate that 12/15-LO deficiency is sufficient to protect from ω6-enriched HF-diet induced steatohepatitis and, as we have shown previously (41), total body insulin resistance.

Published rodent studies have shown that fish oil-based, ω3-enriched HF diets do not induce insulin resistance and adipose tissue inflammation as do isocaloric ω6-enriched HF diets (same % calories from fat) [33], [34], [35]. Although reducing the dietary ω6/ω3 ratio in our current study prevented development of liver inflammation and steatosis, it did not prevent development of insulin resistance (HOMA-IR). The beneficial effect of ω3 fatty acid supplementation on insulin sensitivity in other rodent HF diet-induced models may have been potentiated by three factors, 1) high ratio of ω6/ω3 used in the “control” HF diets, 2) megadoses of EPA+DHA in the fish oil HF diets, and 3) reduced weight gain and food intake observed in mice consuming fish oil-containing vs. standard HF diet. The ω6/ω3 ratios of HF control diets used in several other studies are extreme, ranging from 34∶1 to 86∶1 [17], [18]; in contrast, we used a ratio of 11∶1, which is well within the estimated dietary ω6/ω3 ratio range found in the typical Western-style human diet (10-20∶1) [5]. Based on our calculations using their Methods sections, other rodent studies have used extremely high EPA+DHA (long chain, anti-inflammatory ω3) doses of 1660-4370 mg/kg/day [16], [36], at least 40–104X the common therapeutic dose for an 80 kg human taking a standard prescription of Lovaza fish oil (Figure 1). The EPA+DHA dose in our study was 378 mg/kg/day, approximately 9X the dose that an 80 kg human would consume taking a standard prescription of Lovaza fish oil (Figure 1). In order to minimize weight gain differences commonly attributable to reduced fish oil diet intake, we prepared both our HFH and HFL diets in pelleted form and with added bacon flavoring and anti-oxidants to mask the fragrance of the fish oil in the HFL diet. Thus, in our study, food intake and weight gain exhibited by mice in the HFL low ω6/ω3 ratio group was not reduced. In fact, HFL group mice consumed significantly more calories per day and tended to gain more weight than HFH controls. Rodents in other fish oil diet studies [17], [18], [19], [20], [37] where the fish oil-fed groups consumed less calories and had reduced body weight gain compared to controls may have experienced long-term insulin sensitizing effects that were secondary to reduced weight gain, as has been previously suggested [38]. Thus, the effects of ω3 supplementation on insulin resistance and glucose metabolism remain unclear.

Although it is known that inflammation mediated by the innate immune response plays a critical role in obesity and NAFLD, the role and importance of adaptive immune system is just beginning to be unraveled. Among adaptive cells of the immune system, T lymphocytes play a major role in the inflammatory response. Thus, we hypothesized that an unregulated function of T cells might be critical for NASH. Even though NASH is not considered a Th1-polarized disease, several studies have shown that an excessive production of Th1 proinflammatory cytokines and a deficit of anti-inflammatory cytokines are components in its development [39]. CCR7, the receptor for the chemokine CCL19, is expressed on naïve T-cells, central memory T cells and Treg cells among others. CCR7 regulates trafficking and homing of lymphocytes to and within secondary lymphoid organs and nonlymphoid tissues. Lack of CCR7 expression results in accumulation of conventional T cells in diverse tissues and immediate effector function. Furthermore, it has been demonstrated that IFN-γ is exclusively produced by CCR7- T cells [40]. Herein, we report that HF diet with a high dietary ω6/ω3 ratio significantly reduced the mRNA expression of Ccr7 and increased the expression of its ligand Ccl19, suggesting that T cells are attracted to the liver and once within it, the microenvironment triggers their differentiation into CCR7- cells and retention in the liver. Interestingly, this effect was significantly blunted in mice fed a HF diet with decreased ω6/ω3 ratio or with 12/15-LO deficiency. Lowering dietary ω6/ω3 ratio may be of clinical relevance for reducing T-lymphocyte homing to the liver.

A role for IL-18 has been postulated in the development of hepatic steatosis since both serum IL-18 concentration and hepatic Il18 mRNA expression are elevated in lipopolysaccharide-treated leptin deficient ob/ob mice and high fat diet fed-C57BL/6 mice [41]. Proinflammatory cytokines IL-18 and IL-12 are closely related and act synergistically since the administration recombinant proteins IL-12 and IL-18 concurrently induces the production of elevated levels of IFN-γ and TNF-α in mice [41]. Interestingly, exogenous administration of IL-18 with IL-12 to BALB/c mice induces fatty liver in an IFN-γ dependent manner [42], suggesting that both cytokines play an important role in the inflammatory cascade leading to NAFLD. Different experimental evidences point to an important role of IFN-γ and TNF-α in hepatic damage. The important role ascribed to IFN-γ in hepatic damage is based on its capability to prime Kupffer Cells upon the co-recruitment of monocytes and lymphocytes into the inflamed liver. Also, TNF-α contributes to insulin resistance and hepatic steatosis in diet induced obesity [43]. Accordingly, in the present study we have shown that both increased levels of Il12a, Il12b, Il18, Ifng, Tnfa, and Il10 mRNA expression seen in the liver of HFH fed mice and hepatic steatosis were reduced in HFL fed mice.

Omega-6 and ω3 polyunsaturated fatty acids are *essential* dietary components in mammals and they compete for the same enzymes for their processing, thus, the distribution of these polyunsaturated fatty acids and their metabolite products in plasma and tissue is directly influenced by dietary intake [5], [6]. It has been shown that the positive effects of ω3 fatty acids are connected to modulation of eicosanoid metabolic and signaling pathways, which leads to alteration of polyunsaturated fatty acid subtypes, inflammatory responses, and direct effects on gene expression. Rodents and humans consuming diets rich in ω3 fatty acids, as a result, have reduced production of arachidonic acid and eicosanoid oxidation products of arachidonic acid including prostaglandin E_2_, leukotriene B4, and HETEs [44]. Interestingly, when HETE is added to macrophages, it can cause inflammation by up-regulating proinflammatory gene expression [45]. We have previously demonstrated that 12/15-LO knockout in mice counteracts the HF diet-induced increase in proinflammatory macrophages and cytokine (IFN-γ, IL-12p40 and TNF-α) and chemokine (CXCL1) proteins in adipose tissue [15]. In this study, we demonstrate that increased mRNA expression of hepatic Ifng and Cxcl2/3 seen in HFH-fed WT mice were diminished in HFH-fed 12/15-LOKO mice. The decreased hepatic Il10 mRNA expression seen in HFL mice compared to HFH mice is not surprising. It has been demonstrated that high levels of IL-18 and IL-12 can induce IL-10 [46]. The production of IL-10 could be a mechanism for attenuating the inflammatory response mediated by IL-12. As a consequence, when IL-12 and IL-18 decreased, the attempt to reduce the proinflammatory microenvironment is no longer needed.

12/15-LO is an enzyme that catalyzes the insertion of molecular oxygen into polyunsaturated fatty acids, including the ω6 arachidonic and linoleic acids and the ω3 EPA and DHA. 12/15-LO products of ω6 substrates are involved in the signaling processes of defense response and inflammation, activating Rac, RhoA, MAP kinases, PKC, JNK and NF-κB [47], [48]. We have shown that 12/15-LO oxidation products activate TLR4 in macrophages [49], [50], suggesting a possible connection between 12/15-LO and TLR4 activation in the pathogenesis of insulin resistance. 12/15-LO has also been implicated with obesity. Expression and activity of 12/15-LO is increased in liver and adipocytes after HF diet consumption in rodents [45], [51]. Previously we showed that 12/15-LO deficiency protected mice from adipose tissue inflammation and, remarkably, prevented systemic insulin resistance induced after 2–4 weeks (short-term) HF diet feeding [15], [28]. Longer-term (8–24 weeks) studies showed similar results and also showed that HF induces 12/15-LO activation and beta cell damage in pancreatic islets, which was prevented in 12/15-LO deficient mice [28]. In the current study, we show that 12/15-LO deficiency offers long-term (15 weeks) protection from ω6-enriched HF diet-induced fatty liver, liver inflammation and lymphocyte homing to the liver. 12- and 15-HETE are 12/15-LO oxidation products of ω6 arachidonic acid and 9- and 13-HODE are 12/15-LO products of ω6 linoleic acid, all of which are pro-inflammatory. Herein, we show that levels of oxidized metabolites of arachidonic acid in plasma were significantly reduced by lowering dietary ω6/ω3 ratio and tended to be lower with 12/15-LO deficiency. Although reducing 12/15-LO activity may seem like a potential therapeutic target for counteracting high dietary ω6/ω3 ratio, a simpler strategy would be to reduce dietary ω6/ω3 ratio which would decrease the levels of ω6-derived pro-inflammatory eicosanoids and increase the levels of ω3-derived anti-inflammatory eicosanoids produced overall by cyclooxygenases, lipoxygenases, and epoxygenases.

In this study we examined novel dietary and therapeutic approaches to reduce inflammation in obesity and metabolic syndrome-related diseases. In particular, NASH is a serious liver condition without any available treatments. These data strongly suggest that either reducing dietary ω6/ω3 ratio or inhibiting the enzymatic activity of 12/15-LO may protect against ω6-rich Western diet-induced steatohepatitis.
